# How leaky is the gut in Parkinson’s disease?

**DOI:** 10.1016/j.ebiom.2025.105796

**Published:** 2025-06-03

**Authors:** Pascal Derkinderen, François Cossais, Kristína Kulcsárová, Matej Škorvánek, Loïc Sellier-Montaigne, Emmanuel Coron, Laurène Leclair-Visonneau, Silvia Cerri, Carolina Pellegrini, Malvyne Rolli-Derkinderen

**Affiliations:** a Nantes Université, CHU Nantes, INSERM, The Enteric Nervous System in Gut and Brain Disorders, Nantes F-44000, France; b Department of Anatomy, Kiel University, Germany; c Department of Neurology, P. J. Safarik University, Kosice, Slovak Republic; d Department of Neurology, L. Pasteur University Hospital, Kosice, Slovak Republic; e Department of Clinical Neurosciences, University Scientific Park MEDIPARK, P. J. Safarik University, Kosice, Slovak Republic; f Unit of Cellular and Molecular Neurobiology, IRCCS Mondino Foundation, Pavia 27100, Italy; g Unit of Histology and Embryology, Department of Clinical and Experimental Medicine, University of Pisa, Italy

**Keywords:** Parkinson’s disease, Intestinal epithelial barrier, Intestinal permeability, Tight junctions, Ussing chamber, Organoids, Confocal laser endomicroscopy

## Abstract

The intestinal epithelial barrier (IEB) plays a critical role in health and disease by regulating the absorption of nutrients, electrolytes and water while preventing gut translocation of pathogens. A compromised intestinal barrier has been reported in Parkinson’s disease (PD) further reinforcing the assumption that PD is a gut-brain axis disorder and suggesting that gut-derived factors may participate in disease development and/or progression. However, the diversity of methodology between existing studies on gut permeability in PD, especially regarding the methods used for the evaluation of the IEB, has led to diverging results and it is definitely too early to draw any definite conclusions. We envision novel approaches, such as intestinal organoids and confocal laser endomicroscopy that could be used to study more precisely the IEB in PD.

## Introduction

An accumulating body of literature has emerged in the past 30 years to show that Parkinson’s disease (PD) is not only disorder of the brain but also of the gastrointestinal (GI) tract and more broadly speaking of the gut-brain axis. GI symptoms occur in almost every PD patient at some point^1^^,^^2^ and constipation may precede the appearance of motor symptoms by many years.^3^ Additionally, histopathological studies have consistently reported the presence of alpha-synuclein aggregates, the pathological hallmark of the disease, in *post mortem* intestinal samples^4^^,^^5^ as well as in intestinal biopsies.^6^, ^7^, ^8^ Based on the topographic distribution of alpha-synuclein aggregates established after autopsy from selected cases of patients with PD, Braak and co-workers hypothesized that an unknown neurotropic pathogen could enter the GI mucosa, cross the intestinal barrier, reach the nearby enteric neurons to induce alpha-synuclein aggregation and subsequently reach the central nervous system via the vagus nerve.^9^ This hypothesis, which suggests that the gut is the initial site of PD pathology, is further supported by recent imaging findings that showed dysfunction of enteric vagal innervation in PD.^10^^,^^11^ A prerequisite of the Braak model is that the intestinal barrier should be porous enough to give way to a neurotropic pathogen and this logically prompted several groups to investigate the intestinal permeability in PD and more broadly to study the gut-brain axis and microbiota-gut-brain axis in PD. The microbiota-gut-brain-axis refers to the bidirectional communication between the central nervous system and the GI tract. Apart from the brain, the main components of the microbiota-gut-brain axis include gut microbiota, intestinal epithelium and barrier, enteric nervous system, immune system of the gut and of connections between the gut and the brain including neural connections and humoral components.^12^ Although this concept is still evolving and some data are preliminary, it is suggested that the vast majority if not all players of the microbiota-gut-brain axis are affected in PD (reviewed in^13^^,^^14^) and that they are critically linked to transmit the pathological process from the gut to the brain, as proposed in the body-first subtype of PD.^15^^,^^16^

Unlike other components of the gut-brain axis in PD, such as gut microbiota and the enteric nervous system, the intestinal epithelial barrier (IEB) has been relatively overlooked. In addition, the existing studies on IEB permeability have had diverging results. The aim of this review is therefore to discuss objectively the arguments in favour or against a compromised intestinal barrier in PD. Here, after introducing the basic organization of the intestinal barrier and the methods used to evaluate its functions in human, we discuss in depth, according to the methodologies used, the existing studies on gut permeability in PD and propose directions for future research. We will focus primarily on the IEB; for a more global approach on the possible role of the gut-brain axis in PD, we refer the interested reader to recent reviews on the subject.^13^, ^14^, ^15^^,^^17^

## Intestinal epithelial barrier organisation and evaluation methods: a quick reminder of what neurologist should know

**Fig. 1:**
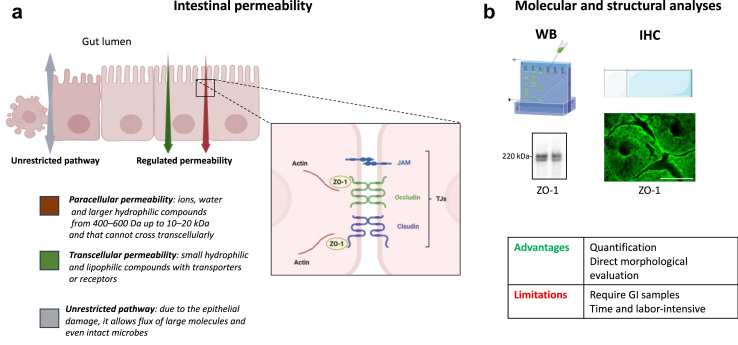
**Morphology and functions of the intestinal epithelial barrier (IEB). (a)** Intestinal permeability is defined as the passage of molecules and ions from the gut lumen to the internal environment. There are three routes for such a passage: one non-specific due to a damaged epithelium (therefore called unrestricted pathways) and two specific routes, one across the plasma membrane of the epithelial cells (transcellular route) and the other one across protein networks called tight junctions (TJs) between two adjacent epithelial cells (paracellular route). Structurally, TJs are formed by a continuous mesh-like network of protein surrounding the apex of epithelial cells, which connects and tightens the space between adjacent cells. They consist of proteins including occludin, claudins, zonula occludens-1 (ZO-1) and junctional adhesion molecule (JAM) **(b)** the expression levels and the subcellular localisation of the TJs components can be analysed by western blot (WB) and immunohistochemistry (IHC). An example of western blot for the detection of ZO-1 in colonic biopsies (controls subjects), with a doublet migrating at 220 kDa (two isoforms) is shown. IHC performed in colonic mucosa (biopsy) from one control subject shows a typical distribution of ZO-1 with a honeycomb pattern (scale bar is 100 μm). Created in part with BioRender.com and with Servier Medical Art, licenced under the Creative Commons Attribution 3.0 Unported Licence.

The intestinal epithelium is a single layer of cells lining the gut lumen that constitutes the largest and most important barrier against the external environment; it allows the passage of nutrients, electrolytes and water and concomitantly prevents gut translocation of pathogens (see description in^18^). This barrier function is primarily maintained via protein networks called tight junctions (TJs) located between two adjacent cells and sealing the intercellular space^19^ (Fig. 1a and Panel 1).

Panel 1 **Tight junctions (TJs) are multiprotein complexes that form a seal between adjacent intestinal epithelial cells, thereby maintaining the integrity and regulating the permeability of the IEB.20**

Many molecular components of TJs have been identified and characterised.^19^^,^^20^ Among these, zonula-occludens, claudins and occludin are key components of TJs and have been by far the most studied.

- Zonula occludens-1 (ZO-1) was the first TJs protein to be identified. ZO-1 is an intracellular protein that form a scaffold between transmembrane proteins, such as claudins and occludin, and the actin cytoskeleton.^21^ Two related smaller proteins, ZO-2 and ZO-3, have similar cellular distribution and functions than ZO-1.
- Claudins are a family of membrane-associated proteins, with 27 members identified so far. In contrast to claudins, there is only one main isoform of occludin.^22^^,^^23^ Both claudin and occludin possess four transmembrane domains and two extracellular loops, which are involved in the structural integrity of TJs. The C-terminus tail of both proteins is located intracellularly where it interacts with zonula-occludens proteins, which in turn binds to the actin cytoskeleton, thereby reinforcing TJs anchoring.^22^^,^^23^

The existing studies on the expression levels of TJs in the PD gut have focused on ZO-1, claudin-1 and 4, and occludin.

**Fig. 2:**
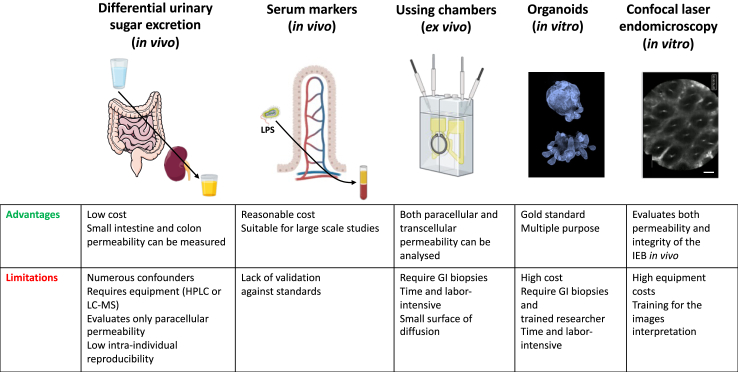
**Current techniques for the evaluation of intestinal permeability.** IEB permeability is classically assessed using 3 main approaches: *in vivo* gut wall permeability tests, *in vivo* detection of translocation markers and *ex vivo* mucosal permeability tests with Ussing chambers. The advantages and limitations of each method are mentioned. Intestinal organoids and confocal laser endomicroscopy (CLE) are two promising additional approaches. Generated from biopsies, organoids allow the amplification of intestinal epithelial barrier in 3-dimensional structure and evaluation of intestinal epithelial barrier functions. The image shows human colonic organoids with a nuclear staining. CLE is a newly developed endoscopic technique that enables *in vivo* microscopic imaging of the gut mucosal layer together with intestinal permeability evaluated by fluorescein leakage. The image shows a colonic mucosa from a rat with chemically-induced colitis; fluorescein leakage is observed at the bottom of the crypts (white dots); scale bar is 20 μm. HPLC: high performance liquid chromatography. LC/MS: liquid chromatography–mass spectrometry. Created in part with BioRender.com and with Servier Medical Art, licenced under the Creative Commons Attribution 3.0 Unported Licence.

There are three main routes for the passage of molecules across the gut epithelium: across the plasma membrane of the epithelial cells (transcellular route) or across TJs between the epithelial cells (paracellular route)^19^ (Fig. 1a). A third, unrestricted, non-specific and TJs-independent pathway can be observed in the presence of epithelial damage (Fig. 1a). The IEB can be assessed in human subjects either functionally or morphologically (Fig. 1, Fig. 2). The most common method for the *in vivo* functional assessment of intestinal permeability is the urinary recovery of orally ingested non-metabolized sugar probes. When the IEB is altered, a higher amount of these markers is transported across the epithelium and subsequently detected in the urine (Fig. 2).^24^ Classically, two different sugars, one monosaccharide and one disaccharide, are ingested in parallel as the measurement of the excretion ratio of these two sugars can correct for confounders such as renal function and intestinal transit time. For example, small intestinal permeability is commonly evaluated by the lactulose/mannitol ratio as the disaccharide lactulose crosses the IEB in a regulated manner through a paracellular route, while mannitol (a monosaccharide) is absorbed by non-mediated diffusion through enterocytes.^24^ Lactulose is degraded by colonic bacteria and is therefore classically replaced by sucralose, which is resistant to bacterial fermentation for the evaluation of colon permeability.^25^ Such a relatively simple and non-invasive *in vivo* approach has nevertheless several limitations, which are listed in Fig. 2.^26^ Despite being more invasive, the *ex vivo* analysis of intestinal samples with Ussing chambers, by allowing the precise analysis of para- and transcellular permeability, is still regarded as the gold standard for the evaluation of IEB permeability^26^^,^^27^ (Fig. 2). For the evaluation of intestinal permeability in human, GI biopsies are classically mounted in Ussing chamber that separate the apical (mucosal or luminal) side from the basolateral side of the tissue. Chambers are kept at 37 °C in a circulating water bath and continuously oxygenated for tissue viability. Fluorescent probes are added to the apical chamber, after which basolateral samples are taken over time to measure passage across the tissue.^27^

Both *ex vivo* and *in vivo* tests for intestinal permeability are time consuming, labour-intensive and not readily available in most laboratories (Fig. 2). There is therefore a growing interest in blood biomarkers for the indirect assessment of mucosal integrity, such as lipopolysaccharide (LPS) and zonulin-1. The evaluation of zonulin-1 as a circulating marker for intestinal permeability will nevertheless not be discussed here as most of the commercially available assays are nonspecific casting doubt on studies published so far.^28^^,^^29^ LPS is a component of the outer membranes of most gram-negative bacteria and the measurement of its serum levels has been proposed to be a marker of bacterial translocation. This approach has nevertheless limitations (Fig. 2), the main ones being that LPS has a very short half-life of just a few minutes and can easily contaminate labware requiring LPS-free conditions throughout the dosage process^30^ (Fig. 2). Given these technical limitations in measuring LPS, LPS binding protein (LBP) an acute-phase protein produced in response to LPS and that binds to it has gained interest as an indirect marker of Gram-negative bacteria translocation.^31^

Morphological and structural changes of the IEB are primarily detected by analysing the expression levels and distributions of the main TJs proteins by Western blot, quantitative PCR and immunohistochemistry (Fig. 1b). It should be kept in mind that some TJs proteins, such as occludin are large molecular weight transmembrane proteins that could pose challenges to membrane extraction required for western blot analysis. As such, an optimized protocol with high stringency lysis buffer should be used (see methods in^32^). For TJs immunohistochemistry, either formalin-fixed paraffin embedded^33^ or whole-mount dissected biopsies^32^ can be used.

Historically, increased permeability of the gut barrier, which results from impaired structure and remodelling of TJs, has been associated with primary GI tract disorders especially inflammatory bowel diseases.^34^ Recently, two promising approaches, namely gut organoids and confocal laser endomicroscopy (CLE), have been introduced to evaluate intestinal barrier function and have been evaluated in inflammatory bowel diseases.^35^^,^^36^ So far, they have not been used in PD and therefore they will be discussed in more detail in the perspective section of this review.

## Why is it important to study the IEB in PD?

The IEB is a key component of the microbiota-gut-brain-axis. The concept of a microbiota-gut-brain axis, a bi-directional communication between the brain and the intestine, is recent and still evolving.^12^ Besides the brain, it primarily consists of gut microbiota, intestinal epithelium/IEB, enteric nervous system, immune systems of the gut and of connections between the gut and the brain including neural connections and humoral components^12^ (Panel 2). The two main components of the enteric nervous system, namely the enteric neurons and enteric glial cells together with the vagus nerve, which are all in close contact with the intestinal epithelium, are involved in the regulation of IEB function in human.^38^^,^^39^

Panel 2 **The concept of the gut influencing the brain and vice versa has existed for more than two centuries.37**

The notion of gut-brain axis (extended to a microbiota-gut-brain axis) as currently defined (a two-way communication system between the brain involving neural and humoral mechanisms) is, however, much more recent.^12^ It is still an evolving concept and data on its function and composition are changing rapidly. However, there are well-established players in this bidirectional signalling system:

- The gut microbiome refers to the trillions of bacteria, viruses and fungi that reside in our gut (bacteria are by far the most studied). Data obtained *in vitro* and in animal models showed that gut microbiota and its metabolites could influence IEB permeability.^40^
- In the gut wall, besides the intestinal epithelial cells and the IEB, the enteroendocrine cells are other key players of the gut-brain axis. These hormone-secreting cells are dispersed in the gut epithelial lining; their apical surface is in contact with the gut lumen while their basal portion contains secretory granules.^41^ Such an orientation allows enteroendocrine cells to respond to intraluminal signals such as nutrients or microbiota-derived metabolites and to produce hormones such as ghrelin and 5-hydroxytryptamine, which have a broad range of effects on gut and brain. More recently, enteroendocrine cells were shown to exhibit neuron-like features and to express alpha-synuclein leading to the assumption that they might be involved in the development of PD.^42^ The enteric neurons, which form the intrinsic innervation of the gut, are embedded in the gut wall. They synapse with neurons from the extrinsic innervation of the GI tract, especially vagal neurons and as such are critical components of the gut-brain axis.^43^ Enteric glial cells, which are regarded as the enteric counterpart of central nervous system astrocytes are found accompanying enteric neurons throughout the gut.^44^^,^^45^ The integrity and homoeostasis of both enteric neurons and glial cells are tightly dependent on gut microbiota.^46^^,^^47^
- Finally, but not least, the intestinal tract contains a plethora of immune cells, which represents the largest collection of immune cells in the body.^37^
- The connection between the intestine and the brain is mediated by at least two pathways, one neuronal and one humoral (e.g., bloodstream). Both the sympathetic and parasympathetic pathways (mainly the vagus nerve), drives both afferent signals, arising from the gut lumen, and efferent signals from the brain to the enteric nervous system and gut wall.^43^ Regarding the humoral pathway, hormones, neurotransmitters, intercellular mediators, and cytokines, produced by gut bacteria, enteroendocrine cells and gut immune cells, are released into the bloodstream to reach the brain for instance through the area postrema.^12^

Quite remarkably, there is now mounting evidence to suggest that most if not all of the actors of the microbiota-gut-brain-axis are affected during the course of PD. Patients with PD suffer from gut dysbiosis (reviewed in^48^), which is associated with changes in the amount of microbiota-derived metabolites such as short chain fatty acids in both feces and plasma^49^^,^^50^; recent findings also showed that amyloids produced by gut bacteria, such as enterobacteria, can promote alpha-synuclein aggregation *in vitro*^51^ and exacerbate alpha-synuclein pathologies in *Caenorhabditis elegans* and rodents model of PD.^52^, ^53^, ^54^ In addition, both the enteric neurons and the extrinsic autonomic innervation of the gut are affected by the pathologic process in a large majority of subjects with PD^4^^,^^5^ and *in vivo* imaging studies showed parasympathetic denervation of the GI tract in PD.^10^^,^^55^ Both gut dysbiosis and vagal denervation have been observed not only in subjects with fully developed but also with early-stage PD.^11^^,^^56^, ^57^, ^58^, ^59^ Preliminary findings also suggest that enteric glial cells are affected in PD, with biochemical and morphological features reminiscent of reactive gliosis.^60^, ^61^, ^62^ Compared to other players of the microbiota-gut-brain axis, the IEB has been relatively overlooked. This is nevertheless an important issue not only for a better understanding of the role of the IEB in the development of the disease but also for the management of GI symptoms, which are among the most common and burdensome non-motor manifestations in PD.^63^ For example, a leaky intestinal barrier may allow factors from the gut lumen to affect neurons in both the intestine and in the brain thereby impacting disease progression. Moreover, IEB alterations, which have been described in subjects with irritable bowel syndrome may also contribute to the irritable bowel syndrome-like symptoms observed in one fourth of patients with PD, such as abdominal pain, discomfort, constipation, gas, and bloating.^64^ Additionally, the assessment of IEB permeability could be used as a disease biomarker either for early detection or management of disease progression or for a better disease classification. Regarding disease classification, an impaired intestinal barrier would potentially support the model recently proposed by Per Borghammer and colleagues, in which pathology might start in the GI tract and the enteric nervous system in a subset of subjects with PD.^15^^,^^65^

## Is the IEB functionally altered in Parkinson’s disease?

So far, four studies used sugar probes absorption in order to evaluate intestinal permeability in PD^66^, ^67^, ^68^, ^69^ (Table 1). In the first study published on the subject, 15 subjects with PD and 15 age-matched controls were included (Table 1). A lower percentage of mannitol in the urine together with an increased lactulose/mannitol ratio was observed in subjects with PD in comparison to controls. Although such an increase in the lactulose/mannitol ratio is usually suggestive of increased small intestinal permeability, it should be here interpreted cautiously as the sole decrease in mannitol excretion may have accounted for the higher observed ratio.^66^ It should be also noted that, even if the results were significant in group analysis, individual results in both groups were highly overlapping. In addition, there was no evaluation of colon permeability in this study^66^ (Table 1). A subsequent study used a similar approach and found an increased lactulose/mannitol ratio in 3 out of 12 subjects with PD^68^ (Table 1). Again, no assessment of colon permeability was performed. One obvious limitation of this study was the absence of a ‘real’ control group, as results of intestinal permeability tests in subjects with PD were compared to normal values from a reference population.^68^ Finally, and in contrast to prior reports, two studies performed by the same research group found no difference in the lactulose/mannitol ratio between subjects with PD and control subjects but observed higher sucralose excretion in subjects with PD^67^^,^^69^ (Table 1). Such a pattern of sugar absorption is suggestive of increased colonic permeability.^67^^,^^69^ In one of these studies, the changes in sugar excretion were observed in untreated subjects with PD suggesting that dopaminergic treatment was not implicated.^67^ It is notable that these two studies had a small sample size and presented the results as bar graphs rather than individual values precluding the appreciation of interindividual variability.^67^^,^^69^ The only study that analysed gut permeability with colonic biopsies mounted in Ussing chamber did not show any difference between subjects with PD and controls either para- or transcellularly.^32^ In addition, no relationship was observed between the amount of enteric alpha-synuclein deposits and IEB permeability.^32^ Overall, the existing studies on the functional evaluation of the IEB in PD have yielded divergent results and no conclusions can be drawn. This can be explained, among other factors, by the relatively small sample size of the studies as well as the different methodologies used.

**Table 1:** Existing studies on the IEB permeability in PD.

Study	PD	C	DD	DA drugs	Technique	GI region	Results PD vs C
Davies et al., 1996^66^	15	15	6	15/15	Sugar probes	SI	↑ L/M Ratio
Forsyth et al., 2011^67^	9	10	2.5	0/9	Sugar probes	SI and colon	No ≠ L/M ratio; ↑sucralose excretion
Salat-Foix et al., 2012^68^	12	0^a^	6.8	10/12	Sugar probes	SI	↑L/M Ratio in 3/12 PD
Clairembault et al., 2015^32^	31	11	9	23/28	Ussing	Sigmoid	No ≠ permeability
Perez-Pardo et al., 2019^69^	6	6	8	4/6	Sugar probes	SI and colon	No ≠ L/M ratio; ↑sucralose excretion

C: controls; DA drugs: numbers of patients with dopaminergic treatment; DD: disease duration; GI region: GI tract region evaluated by the proposed technique. L/M ratio: lactulose/mannitol ratio; No ≠: no difference between PD and C. SI: small intestine.

a Values from PD subjects were compared to a set of values regarded as physiological.

Logically, given the availability of plasma assays, more numerous studies analysed the blood levels of either LPS or LBP in PD. Given the technical issues in measuring LPS in blood with sufficient accuracy, only 3 studies focused on circulating LPS in PD^67^^,^^70^^,^^71^ (Table 2). One small sample size study found no difference between PD and controls,^67^ while two studies with larger populations reported higher LPS levels in PD compared to controls.^70^^,^^71^ It should however be noted that the standard deviation in these two latter studies was especially high and individual results in both groups were highly overlapping. Regarding LBP, the available studies have had somewhat conflicting results (Table 2): 5 studies reported lower LBP blood levels in cases with PD when compared to healthy controls,^67^^,^^69^^,^^72^^,^^73^^,^^75^ whereas the 3 remaining studies found either no difference or even increased plasma LBP in PD.^62^^,^^74^^,^^76^ There is no definitive explanation for the discrepancies between these studies but it underlines the difficulties in interpreting plasma LBP measurements as an increase in LPS could either induce an upregulation of LBP (since the protein is directly secreted in response to LPS) or a decreased level (as LBP binds to LPS, thereby reducing the amount of ‘free’ LBP, which is measured by ELISA kits).

**Table 2:** Existing studies on LPS and LBP blood levels in PD.

Study	PD	C	Unit	LPS C	LPS PD	Technique	Results
Forsyth et al., 2011^67^	9	10	EU/ml	0.82 ± 21	0.84 ± 13	LAL	Mean ± SE, no ≠
Loffredo et al., 2020^70^	8	64	EU/ml	0.15 ± 0.05	0.31 ± 0.05	LAL	Mean ± SD, ↑ in PD
Wijeyekoon et al., 2020^71^	41	41	EU/ml	1.20 ± 0.64	1.91 ± 0.66	LAL	Mean ± SD, ↑ in PD
**Study**	**PD**	**C**		**LBP C**	**LBP PD**	**Technique**	

Forsyth et al., 2011^67^	9	10	ng/mL	84,291 ± 31,380	22,856 ± 5540	ELISA	Mean ± SE, ↓ in PD
Pal et al., 2015^72^	94	99	ng/mL	11,280 ± 7422	9344 ± 6694	ELISA	Mean ± SD, ↓ in PD
Hasegawa et al., 2015^73^	51	36	μg/mL	10.1 ± 5.1	7.8 ± 2.4	ELISA	Mean ± SD, ↓ in PD
Perez-Pardo et al., 2019^69^	5	5	μg/mL	33.47 ± 6.20	15.73 ± 3.75	ELISA	Mean ± SD, ↓ in PD
Aho et al., 2021^74^	55	56	pg/mg	3.38 ± 1.45	3.21 ± 1.26	Multiplex	Mean ± SD, no ≠
Chen et al., 2021^75^	248	149	μg/mL	10.1 ± 3	9.08 ± 2.91	ELISA	Mean ± SD, ↓ in PD
Bellini et al., 2023^62^	13	19	ng/mL	23 ± 2	42 ± 4	ELISA	Mean ± SEM, ↑ in PD
Zhao et al., 2023^76^	352^a^	352	μg/mL	24.7 (16–38)	26.9 (18–41)	ELISA	Median (IQR), no ≠

C: controls; EU: endotoxin unit; IQR: interquartile range; LAL: Limulus Amoebocyte Lysate; SD: standard deviation; SE: standard error. SEM: standard error of the mean.

a All PD cases were prospective. LBP levels in plasma collected at recruitment, which was on average 7.8 years before diagnosis of the PD cases.

## Is the IEB morphologically altered in Parkinson’s disease?

From a morphological perspective, 5 studies analysed the expression of TJs proteins in colonic biopsies of subjects with PD (Table 3). In all of these studies, biopsies were taken from the sigmoid and/or descending colon. In a seminal paper, when colonic biopsies from 31 patients with PD were analysed by Western blot and compared to 11 age-matched controls, a decrease in the expression levels of occludin, but not of ZO-1 was observed in samples from subjects with PD^32^ (Table 3). Additional immunofluorescence experiments showed altered subcellular distribution of both occludin and ZO-1 in the colonic mucosa of 22 out of 31 patients with PD. Such changes were also observed in 5 early untreated patients with PD suggesting that they were unrelated to drug treatment.^32^ It is notable that the decreased expression of occludin in PD samples was not associated with abnormal barrier function,^32^ as already reported in occludin-deficient mice.^79^

**Table 3:** Existing studies on the expression levels of TJs proteins in PD.

Study	PD	C	DD	DA drugs	Results PD vs C
Clairembault et al., 2015^32^	31	11	9	23/28	↓ occludin and claudin-1 (WB)^a^, no ≠ ZO-1 (WB) Altered distribution of occludin and ZO-1 (IF)
Perez-Pardo et al., 2019^69^	6	6/4^b^	8	4/6	↓ ZO-1 (IF) ↑ claudin-1 (chip), no ≠ claudin-2,3,4 (chip) Altered distribution of ZO-1 (IF)
Bellini et al., 2023^62^	10	10	6.3	10/10	↓ claudin-1 (IF)
Ioannou et al., 2024^77^	12^a^	20	UK	N.A	↑ claudin-1 and 4, ↑ ZO-1, ↑ occludin-1 (qPCR) No ≠ claudin-4 (WB) Altered distribution of occludin-1 (IF)
Liao et al., 2024^78^	37	34	2.7	N.A^c^	↓ ZO-1 (IF)

C: controls; chip: mRNA analysis were performed with Affymetrix gene chip; DD: disease duration; DA drugs: numbers of patients with dopaminergic treatment; IF: immunofluorescence; qPCR: quantitative, real-time PCR; WB: Western blot.

a The results from claudin-1 WB were published in Ref.^13^

b Samples from 6 subjects were available for IF, while only 4 were used for gene expression (chip).

c Only mean levodopa equivalent dose was mentioned.

Two studies immunohistochemically analysed the expression levels and the distribution of ZO-1 in the colonic mucosa of subjects with PD.^69^^,^^78^ Both studies found lower expression of ZO-1 in PD samples when compared to controls (Table 3). In addition, decreased ZO-1 expression was associated with a redistribution of the protein (less fluorescence intensity at the apical surface in the crypts) in one of these studies.^69^ Using a similar approach, a decreased expression and altered distribution of claudin-1 was observed in the colonic mucosa of subjects with PD; no other TJs proteins were studied^62^ (Table 3). A bit counterintuitively, a recent study showed that the expression levels of claudin-4, occludin and ZO-1 mRNAs were higher in colonic biopsies of subjects with PD with constipation than in controls.^77^ In addition, structural changes of the colonic crypts were observed in PD subjects with constipation when occludin immunofluorescence was performed^77^ (Table 3). The few available studies therefore seem to indicate that PD is accompanied by structural changes in the IEB but the available data are preliminary and sparse and need to be confirmed in larger and more comprehensive studies.

## Conclusion and perspectives: the IEB in PD, how to move forward?

At first glance, if we consider separately the existing studies on the IEB in PD, it could be tempting to assert that PD is associated with a porous gut during the course of disease, at least in some patients. However, as we have seen, a more detailed analysis of the existing literature does not allow definite conclusions to be drawn, as all of these studies suffer from a number of limitations: (1) most of these studies have been carried out in patients with full-blown PD who received dopaminergic treatment, two possible confounders (2) these studies have yielded conflicting results not only because of their (very) small sample size but also because of the technical issues inherent to the approaches that were used, namely sugar probes absorption and plasma biomarkers (3) morphological analysis was mainly performed by targeting only one or two TJs protein (4) all studies were monocentric (5) clinico-pathological correlations were scarce or absent (6) no longitudinal follow-up was performed and (7) no correlation between morphological and functional changes was performed. It therefore remains to be determined if the IEB is altered in PD even at the early stage of the disease and if gut permeability is associated with a more severe disease progression.

**Fig. 3:**
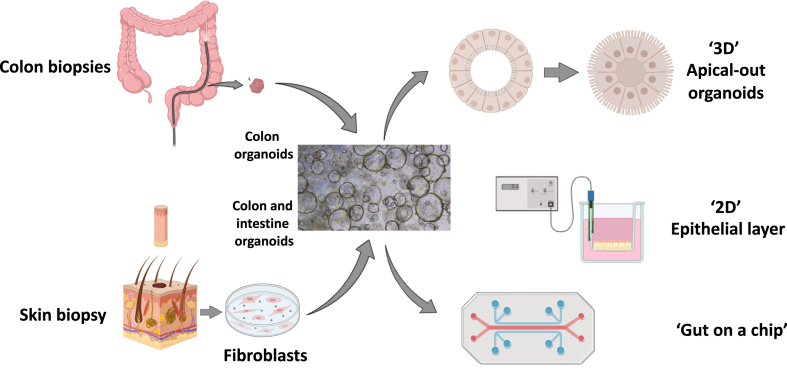
**Potential use and application of human gut organoid technology for the evaluation of intestinal permeability.** Intestinal organoids are derived from either induced pluripotent stem cells or colonic biopsies and form cyst-like structure. Epithelial permeability can be evaluated in 3-D spherical organoids. While these organoids form a closed hollow lumen, it is possible to reverse their polarity. The resulting “apical-out” organoids maintain a barrier function and provide an access to the luminal side.^83^ They allow barrier evaluation for example by measuring the passage of fluorescent probes and the expression levels of TJs genes and proteins. Alternatively, organoids could be cultured as 2-D monolayer in Transwell chambers. Permeability can be studied by measuring either transepithelial electrical resistance or flux of fluorescent markers. Eventually, organoids can be used in gut-on-chip models. These microfluidic devices are made of clear and flexible polymers into which several hollow channels or chambers are moulded. Created in part with BioRender.com and with Servier Medical Art, licenced under the Creative Commons Attribution 3.0 Unported Licence.

So how can we move forward? Based on the limitations of the existing studies, it could be proposed to conduct larger scale multi-centre studies with multiple permeability tests by combining *in vivo* sugar absorption and *ex vivo* permeability tests together with the analysis of the expression levels of a panel of TJs proteins. However, it is unlikely that such an approach will provide a definitive answer. Indeed, until now, a significant and reproducible altered gut permeability, regardless of the method used, has only been observed in disorders with significant digestive inflammation, such as inflammatory bowel diseases.^80^ It is unlikely that such a degree of digestive inflammation and associated intestinal hyperpermeability will be achieved in PD.^81^^,^^82^ Therefore, more sensitive approaches should be used to unmask the existence of a possible altered gut permeability in PD. As such, gut organoids may represent the new gold standard for the investigation of epithelium physiology. Gut organoids can be generated either from routine GI biopsies or from induced pluripotent stem cells (iPSC) differentiated into enterocytes (these can be obtained from skin biopsy-derived fibroblasts) (Fig. 3). These organoids form long-term tridimensional self-renewing structures, which closely mimic the intestinal epithelium^84^ and maintain morphological changes of the IEB under culture conditions.^85^ In addition to epithelial cells, improved models now incorporate both myofibroblasts and neurons, which form a subepithelial tissue.^86^ Once established, gut organoids culture can be cryopreserved and used in different ways for the evaluation of IEB either functionally or morphologically (Fig. 3). More broadly and beyond the sole evaluation of the IEB, gut organoids could be used to help understand the role of the GI tract and the enteric nervous system in the pathophysiology of PD. For example, the ability of potential initiators of alpha-synuclein aggregation such as bacteria and bacterial amyloid proteins^87^ to cross the human intestinal barrier could be studied with apical-out organoids (Fig. 3). The role of the microbiota and the enteric nervous system, two regulatory housekeepers of the intestinal barrier,^88^ which are located respectively on the apical and basal face of the digestive epithelium, could be analysed using co-culture systems (Fig. 3). Despite recent advances, gut organoids still have limitations. Whatever the technique used (either GI biopsies or iPSC), organoids lack immune cells such as lymphocytes and macrophage, which are key components of the gut brain-axis critically involved in IEB homoeostasis (reviewed in^89^^,^^90^). Furthermore, organoids derived from iPSC may still retain some ‘epigenetic memory’ from their original cell type (likely due limitations in reprogramming efficiency), which may limit their utility for the evaluation of IEB function in PD.^91^ Aside from organoids, *in vivo* assessment of IEB integrity has also been successfully achieved using confocal laser endomicroscopy (CLE), a novel endoscopic method that permits on-site microscopy of the GI epithelium.^92^ This is made possible by the combined use of a laser probe (inserted through the working channel of the endoscope), which illuminates the tissue of interest and intravenous infusion of fluorescein that makes the tissue fluorescent.^93^^,^^94^ Such an approach enables the visualization of morphological changes in the gut epithelium^95^ together with functional alterations of the IEB, which are reflected by both fluorescein leakage and the presence of epithelial gaps (these two parameters can be quantified).^35^ One obvious advantage of CLE is that it allows a longitudinal monitoring of both morphology and function of the IEB, as already shown in inflammatory bowel diseases.^35^ Taken together and despite their potential limitations, both organoids and CLE are promising tools not only to determine if the IEB is indeed altered in PD subjects but also to perform clinico-pathological correlations, to improve our understanding in the role of the GI tract in PD and possibly to provide ‘enteric’ parameters in order to monitor disease progression. It should be however kept in mind that, so far both approaches have been mainly used and validated in primary GI disorders, such as inflammatory bowel diseases^35^^,^^85^^,^^96^ and it still remains to be determined whether they will bring relevant insights in neurodegenerative disorders^97^ and in particular in PD.^98^ Finally, a point worth discussing is the IEB as a potential therapeutic target. It has been suggested that the restoration of IEB physiological properties may represent a novel disease-modifying approach in some GI disorders.^99^ In theory, preservation and/or restoration of the IEB can be obtained by modifying the composition of the microbiota (with prebiotics, probiotics or fecal transplants), by using bacterial metabolites such as butyrate, or by a pharmacological approach. In an open label study, synbiotic supplementation (combining pre- and probiotics) improved not only constipation and bowel movements but also numerous non-motor symptoms in subjects with PD^100^ whereas a prebiotic intervention with resistant starch, which induces butyrate production through gut microbiota improved non-motor symptoms including depression but had no effect on bowel habits.^101^ Regarding faecal microbiota transplant, existing studies have had contrasting results. Among the three controlled studies that used changes in global or motor function as primary outcomes, two reported a mild but significant improvement in the treated group^102^^,^^103^ whereas the third study reported no changes in motor function between treated and placebo groups.^104^ These diverging results may be explained by technical differences between studies including among others administration routes of the faecal material, selection of donors and recipients and types of placebos.^104^ Finally, larazotide acetate, a small peptide functioning as a TJs regulator has shown promising results in coeliac disease^105^ and it could be tempting to suggest that this could also be the case in PD. There is no need to mention all that these gut-targeted strategies are hot topics in PD. This is nevertheless an emerging field and, in the context of this review, it remains to be determined if some of the above-mentioned approaches are capable of targeting the IEB. Before going any further, it is essential to clearly demonstrate that the IEB is altered in PD, at least in a subset of subjects and to identify reliable techniques for a longitudinal assessment of the IEB.

## Outstanding questions

There is currently a clear need for more research to determine if the IEB is actually dysfunctional and/or morphologically altered in PD. The existing studies on the IEB in PD have had so far diverging results, which can be explained at least in part by the inclusion of subjects with different disease subtypes, severity or duration. It will be therefore critical to study the IEB in subjects with the recently described body-first PD and in idiopathic RBD. Given the limitations of existing techniques, new approaches could be proposed to help us identify IEB defects in PD, such as confocal laser endomicroscopy and gut organoids. If a defect in barrier permeability and/or structure is eventually identified, therapeutic approaches aimed at maintaining or restoring the IEB could be evaluated as disease-modifying strategies.

**Search strategy and selection criteria.**

We searched PubMed for peer-reviewed articles written in English and published between Jan 1, 2019, and September 30, 2024. We used the search terms “Parkinson’s disease AND intestinal barrier AND human”, “Parkinson’s disease AND leaky gut AND human”, “Parkinson’s disease AND tight junctions AND human”, “Parkinson’s disease AND LPS AND human”, “Parkinson’s disease AND LBP AND human”. We also used older articles (when more recent papers with similar scientific relevance were unavailable). All authors agreed on the final list of references, which was selected on the basis of originality, impact, and topical relevance.

## Contributors

PD: conceptualisation, writing first draft, review, and editing. FC: review and editing. KK: review and editing. MS: review and editing. LSM: tables and figures preparation. EC: reviewing and editing. LLV: reviewing and editing. SC: review and editing. CP: review and editing. MRD: conceptualisation, writing first draft, review, and editing. All authors read and approved the final version of the manuscript.

## Declaration of interests

MS declares honorarium for scientific advisory board activities and lectures for Abbvie, Berlina, Biogen, Boston Scientific, Desitin, International Parkinson and Movement Disorders Society, Krka, Medtronic, Medis, Medison, Stada, TEVA and UCB and reports grants from Slovak Grant and Development Agency, Slovak Scientific Grant Agency, EU Renewal and Resilience Plan and European Regional Development Fund (ERDF) (all paid to the institution). PD reports grants from ANR (Agence nationale de la recherche) and Fondation pour la recherche sur le cerveau (Amadys), all paid to the institution. LLV reports grants from CHU de Nantes and France Parkinson (all paid to the institution). MRD reports grant from ANR (Agence nationale de la recherche), paid to the institution. KK reports funding from the Funding provided by the Slovak Scientific Grant Agency and the Slovak Research and Development (all paid to the institution).
